# A New Aurora in Anaplastic Thyroid Cancer Therapy

**DOI:** 10.1155/2014/816430

**Published:** 2014-07-01

**Authors:** Enke Baldini, Massimino D'Armiento, Salvatore Ulisse

**Affiliations:** Department of Experimental Medicine, “Sapienza” University of Rome, Viale Regina Elena 324, 00161 Rome, Italy

## Abstract

Anaplastic thyroid cancers (ATC) are among the most aggressive human neoplasms with a dire prognosis and a median survival time of few months from the diagnosis. The complete absence of effective therapies for ATC renders the identification of novel therapeutic approaches sorely needed. Chromosomal instability, a feature of all human cancers, is thought to represent a major driving force in thyroid cancer progression and a number of mitotic kinases showing a deregulated expression in malignant thyroid tissues are now held responsible for thyroid tumor aneuploidy. These include the three members of the Aurora family (Aurora-A, Aurora-B, and Aurora-C), serine/threonine kinases that regulate multiple aspects of chromosome segregation and cytokinesis. Over the last few years, several small molecule inhibitors targeting Aurora kinases were developed, which showed promising antitumor effects against a variety of human cancers, including ATC, in preclinical studies. Several of these molecules are now being evaluated in phase I/II clinical trials against advanced solid and hematological malignancies. In the present review we will describe the structure, expression, and mitotic functions of the Aurora kinases, their implications in human cancer progression, with particular regard to ATC, and the effects of their functional inhibition on malignant cell proliferation.

## 1. Aurora Kinases: From Genes to Proteins

**Figure 1:**
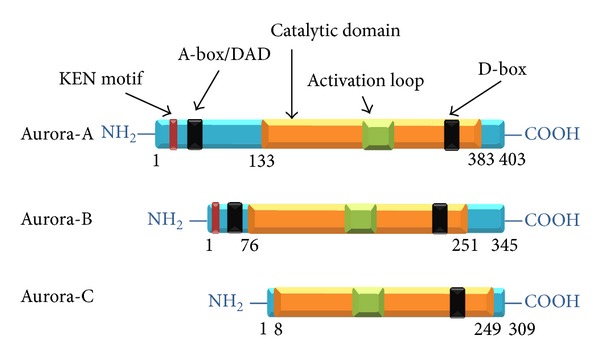
Schematic representation of Aurora kinase proteins. D-box, destruction box; DAD, D-box activating domain; KEN motif, amino acidic K-E-N which serves as targeting signal for the Cdh1-anaphase promoting complex.

The Aurora kinases belong to a family of serine/threonine kinases having in the Ipl1p (increase in ploidy 1) gene, subsequently named Aurora gene, the founding member discovered in the budding yeast* Saccharomyces cerevisiae* during a genetic screening for mutations causing defective chromosomal segregation [1]. In yeast, the Ipl1 remains the only Aurora kinase so far identified, while two Aurora kinases have been found in* Drosophila melanogaster* and in* Caenorhabditis elegans* [2–4]. In mammals, three Aurora kinases have been identified and characterized: Aurora-A, Aurora-B, and Aurora-C [5]. The catalytic domains of these three proteins are highly related in sequence, showing 67–76% identity, but their N-terminal domains have little similarity, which is held responsible for their distinct intracellular localizations, substrate specificity, and functions (Figure 1). In addition, the amino acid sequence of the catalytic domains of Aurora-A, Aurora-B, and Aurora-C is highly conserved across different organisms suggesting its relevance for protein functions and regulation mechanisms across species [5]. The expression of all three human Aurora kinases is cell cycle regulated being low in the G1/S phase and maximal in the G2/M phase. In the next three paragraphs, we will briefly summarize our knowledge regarding the characteristics of the Auroras' encoding genes, their promoter regulation, and protein structure.

### 1.1. Aurora-A

The Aurora-A is encoded by the AURKA gene (also known as AIK, Aurora/IPL1-like kinase; ARK1, Aurora related kinase 1; AURA, AURORA2; BTAK, breast tumor-amplified kinase; PPP1R47, protein phosphatase 1 regulatory subunit 47; STK15, serine/threonine-protein kinase 15; STK6, serine/threonine kinase 6), located at 20q13.2 and consisting of 11 exons (Gene ID: 6790).

The AURKA promoter contains a putative TATA-box at −37 to −14 and two CCAAT-boxes at −101 to −88 and at −69 to −56 (Eukaryotic Promoter Database, Swiss Institute of Bioinformatics). Tanaka and colleagues analyzed the 1.8 kb 5′-flanking region of the Aurora-A gene and found two distinct* cis*-regulatory elements: one positively regulates transcription of the Aurora-A gene, while the other is a cell cycle dependent transcriptional repressor [6]. The former is the 7 bp sequence CTTCCGG, located at −85 to −79 and essential for the transcription of the Aurora-A gene, which is bound by the E4TF1, a ubiquitously expressed ETS family protein. The cell cycle dependent transcriptional repressor is formed by tandem repression elements, consisting of a cell cycle dependent element (CDE) located at −44 to −40 (CGCCC) and a cell cycle gene homology region (CHR) located at −39 to −35 (TTAAA) [6]. It is worth mentioning that, in addition to Aurora-A, the G2/M specific transcription of different genes such as cyclin A, cdc25C, cdc2, polo-like kinase, and others is regulated by similar tandem repression elements [6–9]. Over the last few years, a number of transcription factors capable of modulating the expression of AURKA gene have been identified. These include the p53, the HIF-1, and the INI1/hSSNF5, all reported to negatively regulate the activity of the AURKA promoter [10–12]. Conversely, the transcriptional activity of the AURKA promoter has been shown to increase following the interaction with the ΔEGFR/STAT5 complex in glioblastoma cells [13]. Moreover, the oncogene MYCN, a member of the MYC family of basic helix-loop-helix transcription factors, has been described to bind the AURKA promoter either alone or in complex with the DNA methyl binding protein MeCP2 in the neuroblastoma derived cell line Kelly [14]. EWS-Fli1, a fusion gene resulting from the chromosomal translocation (11; 22, q24; q12), encodes a transcriptional activator capable of promoting cellular transformation in Ewing sarcoma cells. Experimental evidence showed that EWS-Fli1 protein upregulates Aurora-A and Aurora-B expression by binding to regulatory ETS-binding sites located at −84 and −71 bp upstream of the transcription initiation sites in both Aurora-A and Aurora-B promoters [15]. Similarly, it has been suggested that activation of the MAPK in pancreatic cancer cells leads to the transcriptional activation of the AURKA and AURKB promoters via ETS2 transcription factors [16].

The 2.4 kb transcript from AURKA gene encodes a protein of 403 amino acids with a predicted molecular mass of 45.8 kDa (Figure 1). Like all Aurora proteins, it is characterized by a C-terminal catalytic domain containing the activation loop. In the latter, an Aurora kinase signature (xRxTxCGTx) is present in which the autophosphorylation of the Thr288 is required for kinase activation [17]. In addition, the Thr288 is positioned within a protein kinase A (PKA) consensus sequence, and* in vitro* experiments indicated a potential role of PKA in Aurora-A phosphorylation [18, 19]. The phosphatase PP1 has been shown to dephosphorylate and inactivate Aurora-A [19]. The C-terminal located destruction box (D-box), containing the motif RxxLxxG, and the N-terminal A-box/D-box activating domain (DAD), containing the motif RxLxPS, play an essential role in Aurora-A degradation by the anaphase promoting complex/cyclosome- (APC/C-) ubiquitin-proteasome pathway. Aurora-A degradation occurs in late mitosis/early G1 phase, when the D-box is targeted by Fizzy related proteins that transiently interact with the APC, and is hCdh1 dependent [18–21]. In the N-terminal region the amino acidic sequence K-E-N, known as KEN motif, is also present, which serves as targeting signal for Cdh1-APC required also for the degradation of other mitotic proteins such as Nek2 and B99 [22]. However, this does not seem to be crucial for Aurora-A degradation [22].

### 1.2. Aurora-B

The Aurora-B is encoded by the AURKB gene (also known as AIK2; AIM1; ARK2; AurB; IPL1; STK5; AIM-1; STK12), mapped to chromosome 17p13.1, and consisting of 9 exons (Gene ID: 9212).

The AURKB promoter contains three putative CAAT-boxes at −99 to −86, at −66 to −53, and at −30 to −17 (Eukaryotic Promoter Database, Swiss Institute of Bioinformatics). By primer extension two major transcription initiation sites were identified [23]. As for the Aurora-A promoter, also the Aurora-B promoter possesses the CDE and CHR elements, though responsible for the cell cycle regulation of its expression, and several CDE-binding proteins have been identified by means of electrophoretic mobility shift assay and biotin-streptavidin pull-down assay, including the E2F-1, E2F-4, and DP-2 [23]. As above described, the AURKB promoter may be positively regulated by transcription factors such as the ETS2 via ETS-binding sites present in the promoter sequence [15, 16].

The 1.4 kb transcript encodes a protein of 345 amino acids with a predicted molecular mass of 39 kDa (Figure 1) [5]. As described for Aurora-A, also the Aurora-B protein is characterized by a catalytic domain, a C-terminal D-box, and an N-terminal A-box/DAD [18–21]. However, although Aurora-B possesses the same D-box as Aurora-A, it is not degraded by the same ubiquitin ligase but undergoes degradation following its binding to the human proteasome *α*-subunit C8 in a proteasome dependent manner [24].

### 1.3. Aurora-C

The Aurora-C is encoded by the AURKC gene (also known as AIE2; AIK3; ARK3; AurC; SPGF5; STK13), which localizes at chromosome 19q13.43 and consists of 7 exons (Gene ID: 6795).

The AURKC promoter contains a CCAAT-box at −36 to −23 (Eukaryotic Promoter Database, Swiss Institute of Bioinformatics). Expression of AURKC is downregulated by PLZF, a transcriptional repressor, through recruitment to its promoter region, and it is of interest to note that the expression levels of PLZF and AURKC mRNAs display opposite patterns in human cervical and colorectal cancers [25].

The 1.3 kb transcript encodes a protein of 309 amino acids with a predicted molecular mass of 35.6 kDa (Figure 1) [5]. As the other members of the family, also Aurora-C is characterized by a catalytic domain present in the C-terminal region of the molecule (Figure 1). However, differently from Aurora-A and Aurora-B, Aurora-C does not contain the KEN and the A-box/DAD motifs in its N-terminal region, while the C-terminal D-box is present. The mechanism(s) underlying its degradation, however, still remains to be elucidated and represents an interesting area of investigation.

## 2. Expression, Subcellular Localization, and Functions of the Aurora Kinases

The three Aurora kinases play relevant functions during the mitotic phase of the cell cycle [18, 19]. As mentioned, these proteins display distinct intracellular localizations, substrate specificity, and functions during mitosis and their expression and activity are tightly regulated at the transcriptional or posttranscriptional level, through phosphorylation/dephosphorylation and protein degradation [26]. In the next paragraphs, we will briefly discuss the main mitotic functions of the Aurora kinases and we will mention recent reports suggesting their extramitotic functions.

### 2.1. Aurora-A

The expression of Aurora-A is cell cycle regulated, being very low during the G1 phase and starting to accumulate at the centrosome in the late S phase to be maximal at the G2-M transition. During mitosis it localizes at the spindle poles to be inactivated and degraded before cytokinesis, as above mentioned [19, 22]. Aurora-A regulates centrosome separation and maturation, cell mitotic entry, and bipolar spindle construction. Centrosome recruitment of Aurora-A is controlled by the Cep192/Spd-2 (centrosome protein of 192 kDa/spindle defective 2) which is also involved in kinase activation during mitosis [27]. Once on the centrosome, Aurora-A promotes the recruitment to the pericentriolar mass (PCM) of a number of proteins required for proper centrosome maturation and function. These include centrosomin, *γ*-tubulin, LATS2 (large tumor suppressor, homolog 2), TACC3 (transforming acidic coiled coil 3), and NDEL1 (nuclear distribution element-like 1) [19, 22, 28].

**Figure 2:**
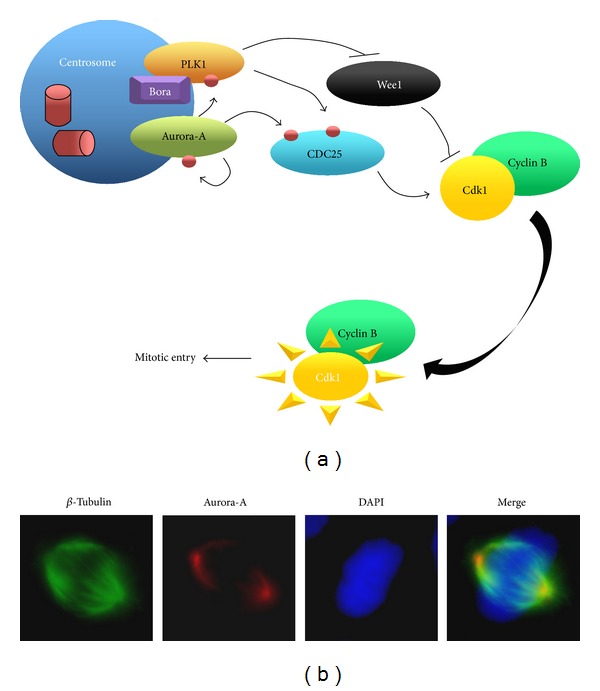
(a) Schematic representation of the pathway induced by Aurora-A to activate the CDK1/cyclin B complex allowing the transition of the cell from the G2 to the M phase. Aurora-A in association with Bora phosphorylates the PLK1. Both Aurora-A and PLK1 phosphorylate CDC25B (cell division cycle 25 B) allowing cyclin dependent kinase 1(CDK1)/cyclin B complex activation and thus promoting mitotic entry. PLK1 facilitates this process also by inhibiting the CDK1 inhibitor WEE1. Inactivation of Aurora-A or Plk1 individually shows no significant effect on Cdk1 activation and entry to mitosis, while their simultaneous inactivation produces a marked delay in both Cdk1 activation and mitotic entry, suggesting that the two kinases have redundant functions. (b) Immunofluorescence showing Aurora-A localization at the spindle pole of an anaplastic thyroid cancer cell in metaphase.

While promoting centrosome maturation, Aurora-A activates the CDK1/cyclin B complex allowing the transition of the cell from the G2 to the M phase [29–31]. In particular, Aurora-A in association with the G2 induced Bora protein phosphorylates the PLK1 (polo-like kinase 1). Both Aurora-A and PLK1 phosphorylate CDC25B (cell division cycle 25 B), a member of the CDC25 family of phosphatases which activates cyclin dependent kinases by removing two phosphate groups, leading to CDK1/Cyclin B complex activation and finally promoting mitotic entry [19, 29–31]. PLK1 facilitates this process also by inactivating the CDK1 inhibitor WEE1 (Figure 2). Inactivation of Aurora-A or Plk1 individually shows no significant effect on Cdk1 activation and entry to mitosis, while their simultaneous inactivation produces a marked delay in both Cdk1 activation and mitotic entry, suggesting that the two kinases have redundant functions [32].

A central role of Aurora-A during mitosis is to support the microtubule-organizer center (MTOC) responsible for the formation of the bipolar spindle. In this context Aurora-A has been shown to form complexes with TACC1 and TACC3, which in turn, by binding to ch-TOG/XMAP215 proteins, stabilize microtubules at the centrosome [33–35]. In addition, Aurora-A interacts with and phosphorylates TPX2, which is capable of promoting spindle microtubule polymerization [22].

### 2.2. Aurora-B

**Figure 3:**
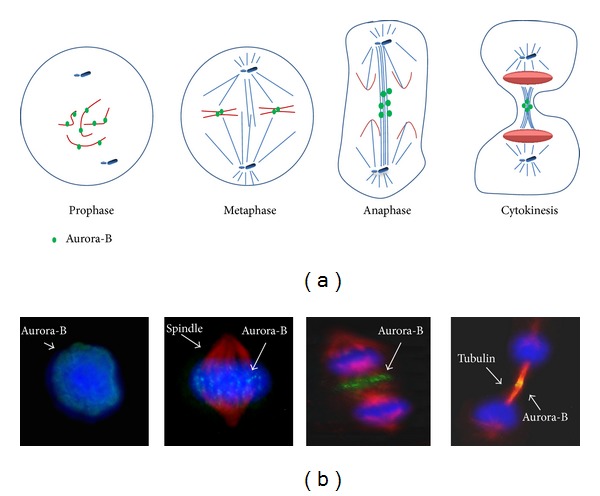
Schematic representation (a) and immunofluorescence images (b) of Aurora-B localization during mitosis in an anaplastic thyroid cancer cell. In (b) Aurora-B is in green, microtubules are in red, and DNA, stained by DAPI, is in blue.

Aurora-B mRNA and protein levels peak at G2/M phase, and the maximum kinase activity is reached from metaphase to the end of mitosis [18, 19]. Aurora-B exerts its action mainly in concert with three other proteins, that is, INCENP (inner centromere protein), survivin, and borealin/Dasra B, with which it associates in the chromosomal passenger complex (CPC). In early prophase, the CPC is located on chromosomal condensing arms where it seems to displace the heterochromatin protein-1 from DNA and to recruit condensin proteins (Figure 3) [36, 37]. From early G2 phase to prophase, Aurora-B phosphorylates histone H3, but its physiological meaning remains unclear. From late prophase to metaphase CPC localizes to the inner centromere, playing a role in formation and stability of the bipolar mitotic spindle, kinetochore assembly, correction of nonbipolar chromosome-spindle attachments, and control of the spindle checkpoint (Figure 3). At the beginning of anaphase CPC relocates to the midzone of the mitotic spindle and to the cell cortex, remaining evident in the midbody of telophasic cells where it modulates the activity of several proteins involved in spindle dynamics, cleavage furrow formation, and completion of cytokinesis (Figure 3) [18, 19, 36, 37].

Aurora-B activation requires its autophosphorylation and binding to INCENP, while all CPC components are necessary for its proper localization during mitosis. Several kinases, such as BubR1 and Bub1 (checkpoint kinases), Mps1 (monopolar spindle 1), Chkl (checkpoint kinase 1), tousled-like kinase-1, Plk1, and TD-60/RCC2 (regulator of chromosome condensation 2), have been recently shown to be involved in Aurora-B activation. TD-60, a protein with a chromosomal passenger-like localization pattern, seems to be also important for centromeric localization of Aurora-B [38]. The phosphorylation status and activity of Aurora-B are modulated by PP1 and PP2A phosphatases; depending on the regulatory subunit they are bound to, these enzymes can directly modulate Aurora-B activity through dephosphorylation or they may dephosphorylate Aurora-B substrates [38].

### 2.3. Aurora-C

Expression of Aurora-C is maximal during the G2/M phase. The observation that Aurora-C is expressed at relative high levels in germ cells during spermatogenesis and oogenesis and at very low levels in somatic cells is of interest. Aurora-C is highly similar to Aurora-B in sequence (61% identity), which may explain why the two kinases display similar localization patterns and share interacting proteins and substrates such as INCENP, survivin, and borealin [18, 39]. Interestingly, when ectopically expressed in cells depleted of Aurora-B, Aurora-C is capable of rescuing the Aurora-B-dependent mitotic functions [40]. It is also worth noting that Aurora-C has been shown to interact with and phosphorylate TACC1 in thyroid cells [40]. In the latter, TACC1 and Aurora-C have been shown to colocalize in the cytokinetic bridge [41].

### 2.4. Extramitotic Functions of Aurora Kinases

Over the last few years, different experimental findings suggest extramitotic functions for Aurora-A and Aurora-C. In particular, Aurora-A has been proposed to affect microtubule dynamics, cell migration and polarity, cilia disassembly, and regulation of intracellular calcium signaling in interphasic cells [22]. Regarding Aurora-C, it has been shown that, along with its interacting protein TACC1, it may be involved in telomere stability. In particular, TACC1 has been shown to bind the LSM7 and SmG proteins involved in telomere formation, while the TFR2 protein, a component of the telomeric complex shelterin, has been shown to form a complex with Aurora-C [42, 43]. Thus, it may be speculated that TACC1 and/or Aurora-C may contribute to telomere homeostasis.

## 3. Aurora Kinases and Cancer

Genetic instability, a hallmark of solid tumors, is thought to represent the mean by which premalignant cells acquire novel functional capabilities responsible for cancer cell growth and tumour progression [44]. In fact, aberrations in chromosome number and structure characterize the majority of human cancers and follow alterations of cellular functions required for appropriate chromosome segregation and integrity of cellular checkpoints [45]. Given the crucial tasks of Aurora kinases in all mitotic stages, their dysfunction and/or dysregulation may well lead to abnormal cell divisions and aneuploidy. However, whether Aurora kinases have a role in cancer initiation is still a matter of debate. It has been reported that the overexpression of either Aurora-A, Aurora-B, or Aurora-C induces cell malignant transformation [46–48]. In different studies, however, although the ability of Aurora-A or Aurora-B to potentiate Ras-induced transformation was demonstrated, the transforming ability of either Aurora-A or Aurora-B overexpression alone was not observed [49, 50].

Aurora-A kinase has been often implicated in cancer progression and its hyperactivation has been demonstrated to induce resistance to microtubule-targeted chemotherapy [51–53]. The AURKA gene is often amplified in many malignancies, and its overexpression has been reported to be significantly associated with a higher tumor grade and a poor prognosis in a number of cancers, such as chondrosarcoma, nasopharyngeal carcinoma, ER-positive breast cancer, glioblastoma, colorectal cancer, gastric cancer, and endometrioid ovarian carcinoma [54–60]. In addition, somatic mutations located within the catalytic domain of Aurora-A, altering kinase activity and subcellular localization, have been described in human cancer cells [61]. The oncogenic potential of Aurora-A derives from a sum of several spatially and temporally distinct actions. Unlike normal cells, in many cancer cells the expression of Aurora-A becomes constitutive throughout the cytoplasm, regardless of the cell cycle phase; this can trigger a plethora of phosphorylated proteins, improper interactions, and mislocalization. Aurora-A may also represent the downstream target of mitogenic pathways, such as MAPK/ERK (mitogen-activated protein kinases), and hence be overexpressed because of their constitutive activation in tumors [52]. The Aurora-A excess interferes with different cell cycle checkpoints, that is, the late G2 checkpoint, which restrains genetically aberrant cells to enter mitosis, the spindle assembly checkpoint, which blocks the metaphase-anaphase transition in cells with defective spindles, and the postmitotic G1 checkpoint, which arrests cell cycle in aneuploid cells [51, 53]. Centrosome amplification and unrestrained multinucleation, leading to abnormal mitotic spindle, are also observed in Aurora-A overexpressing cells [62]. Moreover, Aurora-A may significantly contribute to tumor progression by interacting with and inhibiting several tumor suppressor proteins such as p53, BRCA1 (breast cancer 1), and Chfr (checkpoint with forkhead and ring finger domains).

Aurora-B plays a less clear role in tumorigenesis. An increased level of Aurora-B in normal cells has been shown to induce premature chromosome separation and segregation errors, to promote generation of tetraploid and aneuploid cells, which develop a transformed phenotype* in vitro* and* in vivo*, and, as above mentioned, to potentiate Ras oncogenic activity [47, 50, 63–65]. Neither amplification nor specific mutations of its gene have been shown in tumors; nevertheless, Aurora-B overexpression has been demonstrated in several cancer types, like breast, colorectal, kidney, lung, and prostate cancer, and it has been reported to correlate with the level of genomic instability and with poor prognosis in advanced colorectal cancer, astrocytoma, head and neck squamous cell cancer, and endometrial and hepatocellular carcinomas [51, 63–65]. At present, very little information is available about the role of Aurora-C in cancers. Despite Aurora-C is hardly detected in normal somatic cells, it is highly expressed in various tumor cell lines [66–69]. One study has described the transforming potential of overexpressed Aurora-C in NIH-3T3 cells and a correlation between the level of active kinase and tumor aggressiveness of the cells injected in nude mice [48].

The overexpression of Aurora kinases in human cancers and their relevance in controlling the mitotic process have led to the development of small-molecule inhibitors as putative anticancer drugs. Aurora inhibition results in cytokinesis failure and generation of tetraploid cells, which, depending on the postmitotic checkpoint activation, may be unable to proceed in cell cycle or rather proliferate and become polyploid. The exit from cell cycle is likely to generate viable quiescent cells, whereas endoreplicating cells have greater tendency to undergo apoptosis. Nowadays the Aurora kinase inhibitors are considered a promising therapeutic option, especially against those cancers that do not respond to currently available anticancer therapies [70–77]. About 30 small molecule inhibitors of Aurora kinases are under preclinical and clinical evaluation with some of them, reported in Table 1, undergoing phase I-II clinical trials for solid and hematological cancers [70–77]. The responses obtained in these clinical trials showed either disease stabilization or less frequently partial responses in patients with solid cancers, while more promising activity has been observed in patients with hematological malignancies [70–77]. However, a number of side effects, most of which reversible upon drug withdrawal, were observed [74]. On-target toxicity encountered in the different clinical trials included grade 3/4 neutropenia, leukopenia, and myelosuppression, while off-target effects included hypertension, somnolence, mucositis, stomatitis, proctalgia, grade 3 increase in aspartate aminotransferase, and ventricular dysfunction [70–77]. Cardiotoxicity, associated with death of one patient, has been recorded in a phase II clinical trial with tozasertib (VX-680) [74].

## 4. Thyroid Cancers

Epithelial thyroid cancer (TC) accounts for about 1% of all human tumors and represents the most common endocrine malignancy, the fifth most common cancer in women in the United States [78, 79]. The majority of TC (90–95%) is differentiated carcinomas (DTC), occurring as papillary (PTC) or follicular (FTC) histotypes, the incidence of which has been increasing over recent years [80]. Following dedifferentiation DTC are assumed to generate the poorly DTC (PDTC) and the highly aggressive and invariably fatal anaplastic thyroid carcinomas (ATC) [81, 82]. Relevant molecular alterations encountered in DTC progression comprise gene rearrangements of tyrosine kinase receptors, such as the RET/PTC and NTRK1 (neurotrophic receptor-tyrosine kinase 1), or activating point mutations of proteins mediating cellular responses to growth and differentiation signals, including RAS, BRAF, phosphatidylinositol 3-kinase (PI3K), or the oncogenic fusion protein PAX8-PPAR*γ*, as well as inactivating mutations in the tumor suppressor phosphatase and tensin homolog (PTEN) and TP53 [83–85]. The conversion of early-stage thyroid tumors to more aggressive and invasive malignancies occurs through an epithelial-to-mesenchymal transition (EMT), which implies the loss of cell-cell contacts, remodeling of cytoskeleton, and the acquisition of a migratory phenotype [86, 87]. In fact, abnormal expression of integrins, Notch, MET, TGF*β*, NF-*κ*B, PI3K, TWIST1, matrix metalloproteinases (MMP), components of the urokinase plasminogen activating system (uPAS), and p21-activated kinase (Pak), involved in the EMT, has been identified in PTC progression [86–91]. Genomic instability, a hallmark of solid tumors including TC, is thought to represent the driving force responsible for the generation and accumulation in the malignant cells of the above-mentioned molecular alterations [44, 92–94]. According to this, hypothesis is the evidence that the number and the frequency of chromosomal abnormalities, observed during thyroid cancer progression, increase from the DTC to the PDTC and ATC [92–94].

While the prognosis for DTC patients is favorable, with 10-year survival rate of about 90%, that for patients affected by PDTC and ATC is very poor, with a median survival of only few months from diagnosis [94–96]. It has to be noted, in fact, that the surgical resection of the tumor mass is not effective in ATC patients and treatment of recurrent or persistent PDTC and ATC with conventional radiotherapy and/or chemotherapy provides little or no benefit. Therefore, novel therapeutic approaches are sorely needed for these neoplasms [85, 97].

## 5. Aurora Kinases and Thyroid Cancers

Normal human thyrocytes express all three Aurora kinases in a cell cycle dependent manner [67]. The expression of Aurora-A and Aurora-B in this cell type is mainly regulated at the transcriptional level, while that of Aurora-C appears to be modulated at the posttranscriptional level [67]. An increased expression of all three Aurora kinases has been shown in various cell lines originating from different epithelial thyroid tumor histotypes, compared to normal thyrocytes, as well as in DTC and ATC tissues, compared to normal matched tissues [33, 67, 98]. In addition, a study aimed at evaluating the gene expression profile in ATC, by means of tissue microarray and immunohistochemistry, identified AURKA as one of the most frequently and most strongly overexpressed genes in these tumors [99]. This is consistent with the observation that gain of chromosome 20q, where AURKA is located (20q13.2), is frequently encountered in ATC [100]. Based on these findings, the potential therapeutic value of Aurora kinase inhibition on the proliferation and growth of ATC cells has been evaluated in preclinical studies [101–105]. In particular, the* in vitro* effects of different pan-Aurora kinase inhibitors, including the MK-0457 (VX-680), the SNS-314 mesylate, and the ZM447439, have been investigated on proliferation, apoptosis, cell cycle, ploidy, and anchorage-independent growth of a panel of ATC-derived cell lines [102–104]. These molecules were found to inhibit proliferation of ATC cells in a time- and dose-dependent manner and to impair cancer cells colony formation in soft agar. Cytofluorimetric analysis of cell cultures exposed to the pan-Aurora kinase inhibitors revealed an accumulation of tetra- and polyploid cells because of endoreplication events followed by the activation of caspase-3 and accumulation of a sub-G0/G1 cell population, both indicative of apoptosis [102–104]. Treated cells showed mitotic alterations consistent with the inhibition of Aurora kinases, including major impairment of centrosome functions, with abnormal spindle formation characterized by the presence of short microtubules, inhibition of histone H3 phosphorylation, and inability to complete the cytokinesis. In addition, the selective inhibition of either Aurora-A or Aurora-B has been investigated [101, 105, 106]. The selective inhibition of Aurora-B expression by means of RNA interference, or of Aurora-B function by AZD1152, has been reported to significantly reduce growth and tumorigenicity of ATC-derived cells, both* in vivo* and* in vitro* [101]. Similarly, functional inhibition of Aurora-A by MLN8054 in a panel of ATC-derived cell lines has been shown to inhibit cell proliferation and to induce cell cycle arrest and cell apoptosis [105]. In xenograft experiments, the Aurora-A inhibitor was found to reduce tumor volume by 86% [105]. Interestingly, the combined treatment with MLN8054 and bortezomib, targeting the ubiquitin-proteasome system, showed additive effects on ATC-derived cell proliferation and apoptosis, compared to monotherapy [106]. Pazopanib, an inhibitor of kinases including the VEGFR (vascular endothelial growth factor receptor) shown to have impressive therapeutic activity in patients affected by radioactive iodine-refractory DTC, was tested in a phase II clinical trial on ATC patients [107, 108]. Despite several pazopanib treated ATC patients showed a transient disease regression, no RECIST (response evaluation criteria in solid tumors) response was obtained [106]. More recently, in a preclinical study on a panel of ATC derived cell lines, pazopanib was found to potentiate the cytotoxic effects of paclitaxel* in vitro* and in xenograft experiments [109]. The effects of this pazopanib were attributed to an unexpected off-target inhibition of Aurora-A in ATC derived cell lines. In fact, the same results were obtained when combining paclitaxel and MLN8237, a selective Aurora-A inhibitor. In the same study, the authors also showed that the combined administration of pazopanib and paclitaxel attained a marked and durable regression of lung metastasis, in a single ATC patient [109].

In conclusion, the preclinical and clinical data so far available indicate that Aurora kinase inhibitors may have a therapeutic potential for ATC treatment either in monotherapy or, more likely, in combination therapy with anti-microtubule drug.

## Figures and Tables

**Table 1:** Aurora kinase inhibitors in clinical trials.

	Inhibitor (company) commercial name	Clinical trial
Pan-Aurora inhibitors	VX-680/MK-0457 (Vertex/Merck) Tozasertib	Phase II (terminated due to severe toxicity)
	PHA-739358 (Pfizer/Nerviano) Danusertib	Phase II
	PHA-680632 (Pfizer/Nerviano)	Phase II
	CYC-116 (Cyclacel)	Phase I
	SNS-314 (Sunesis)	Phase I
	R763 (Rigel)	Phase I
	AMG-900 (Amgen)	Phase I
	AT-9283 (Astex)	Phase II
	PF-03814375 (Pfizer)	Phase I
	GSK1070916 (GlaxoSmithKline)	Phase I

Aurora-A inhibitors	MLN8237 (Millennium)	Phase II
	EMD-2076 (EntreMed)	Phase II
	MK-5108 (Vertex)	Phase I

Aurora-B inhibitors	AZD1152 (AstraZeneca)	Phase II
